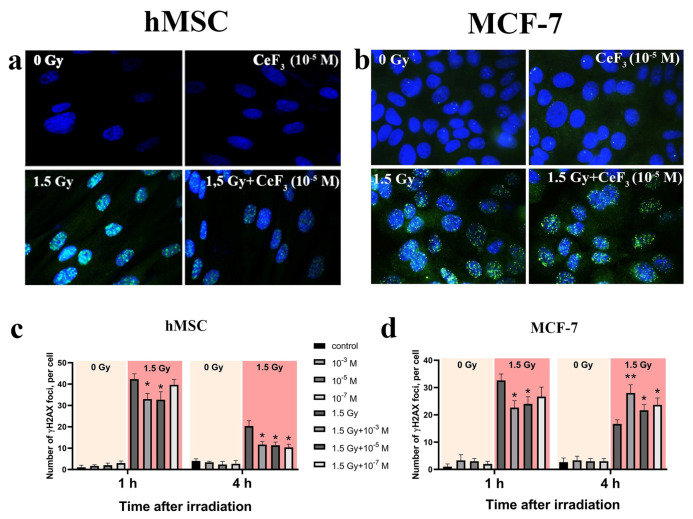# Correction: Chukavin et al. Redox-Active Cerium Fluoride Nanoparticles Selectively Modulate Cellular Response against X-ray Irradiation In Vitro. *Biomedicines* 2024, *12*, 11

**DOI:** 10.3390/biomedicines13081966

**Published:** 2025-08-13

**Authors:** Nikita N. Chukavin, Kristina O. Filippova, Artem M. Ermakov, Ekaterina E. Karmanova, Nelli R. Popova, Viktoriia A. Anikina, Olga S. Ivanova, Vladimir K. Ivanov, Anton L. Popov

**Affiliations:** 1 Institute of Theoretical and Experimental Biophysics, Russian Academy of Sciences, Pushchino 142290, Russia; chukavinnik@gmail.com (N.N.C.); kristina.kamensk@mail.ru (K.O.F.); ao_ermakovy@rambler.ru (A.M.E.); silisti@bk.ru (E.E.K.); nellipopovaran@gmail.com (N.R.P.); viktoriya.anikina@list.ru (V.A.A.); 2 Scientific and Educational Center, State University of Education, Moscow 105005, Russia; 3 Frumkin Institute of Physical Chemistry and Electrochemistry, Russian Academy of Sciences, Moscow 119071, Russia; runetta05@mail.ru; 4 Kurnakov Institute of General and Inorganic Chemistry, Russian Academy of Sciences, Moscow 119991, Russia; van@igic.ras.ru

In the original publication [1], there was a mistake in Figure 4 as published. Due to a technical error, in Figure 4b, an incorrect image labeled “CeF_3_ (10^−5^ M)” was used. The corrected Figure 4 appears below. The authors state that the scientific conclusions are unaffected. This correction was approved by the Academic Editor. The original publication has also been updated.